# Harnessing three-dimensional porous chitosan microsphere embedded with adipose-derived stem cells to promote nerve regeneration

**DOI:** 10.1186/s13287-024-03753-w

**Published:** 2024-06-01

**Authors:** Yaqiong Zhu, Dan Yi, Jing Wang, Yongyi Zhang, Molin Li, Jun Ma, Yongjiao Ji, Jiang Peng, Yuexiang Wang, Yukun Luo

**Affiliations:** 1 Department of Ultrasound, the First Medical Centre, Chinese PLA General Hospital, Beijing, 100853 China; 2 Medical School of Chinese PLA, Beijing, 100853 China; 3 Beijing Key Lab of Regenerative Medicine in Orthopaedics, Chinese PLA General Hospital, Beijing, China; 4 Key Lab of Musculoskeletal Trauma & War Injuries, Chinese PLA General Hospital, Beijing, China; 5 Beijing Key Laboratory of Chronic Heart Failure Precision Medicine, Chinese PLA General Hospital, Beijing, China; 6 Department of Orthopaedic Surgery, The First Affiliated Hospital of University of Science and Technology of China, Hefei, Anhui Province China; 7 Department of Rehabilitation Medicine, the Second Medical Centre, Chinese PLA General Hospital, Beijing, China; 8 No.962 Hospital of the PLA Joint Logistic Support Force, Harbin, China; 9 Department of Orthopaedics, The Fourth Centre of Chinese PLA General Hospital, Beijing, China

**Keywords:** Adipose-derived stem cells, Chitosan microsphere, Nerve regeneration, Nerve guide conduits, Cell therapy

## Abstract

**Background:**

Nerve guide conduits are a promising strategy for reconstructing peripheral nerve defects. Improving the survival rate of seed cells in nerve conduits is still a challenge and microcarriers are an excellent three-dimensional (3D) culture scaffold. Here, we investigate the effect of the 3D culture of microcarriers on the biological characteristics of adipose mesenchymal stem cells (ADSCs) and to evaluate the efficacy of chitosan nerve conduits filled with microcarriers loaded with ADSCs in repairing nerve defects.

**Methods:**

In vitro, we prepared porous chitosan microspheres by a modified emulsion cross-linking method for loading ADSCs and evaluated the growth status and function of ADSCs. In vivo, ADSCs-loaded microcarriers were injected into chitosan nerve conduits to repair a 12 mm sciatic nerve defect in rats.

**Results:**

Compared to the conventional two-dimensional (2D) culture, the prepared microcarriers were more conducive to the proliferation, migration, and secretion of trophic factors of ADSCs. In addition, gait analysis, neuro-electrophysiology, and histological evaluation of nerves and muscles showed that the ADSC microcarrier-loaded nerve conduits were more effective in improving nerve regeneration.

**Conclusions:**

The ADSCs-loaded chitosan porous microcarrier prepared in this study has a high cell engraftment rate and good potential for peripheral nerve repair.

**Graphical Abstract:**

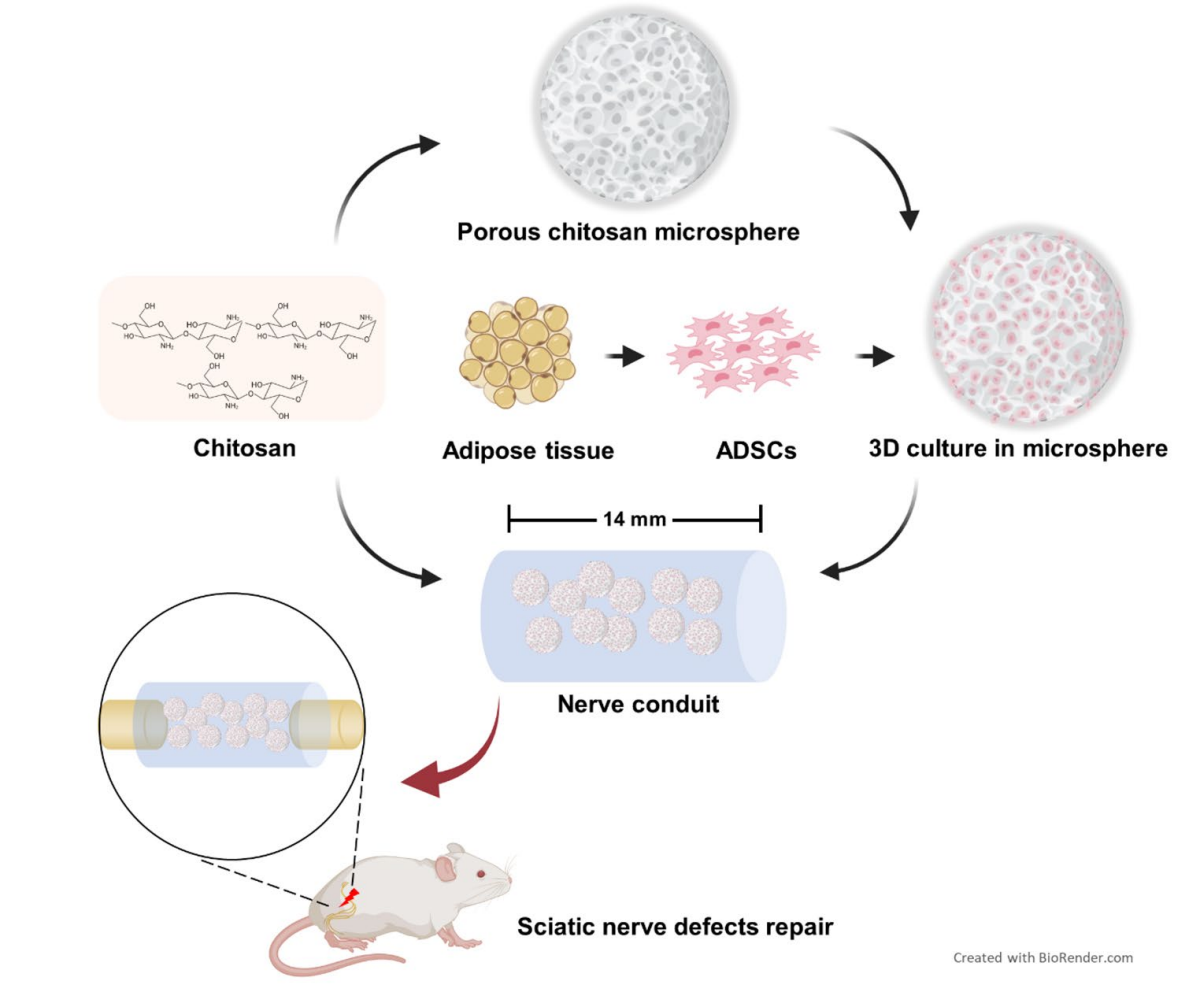

## Background

Peripheral nerve injury (PNI) is very common in clinical practice, with more than 50,000 new cases of PNI each year worldwide, mainly caused by traffic accidents, natural disasters, disease, and iatrogenic injury [1]. The peripheral nervous system has a certain regenerative capacity, but when the nerve is severed, or a large gap defect is caused, surgery is usually required to restore its continuity. Autologous nerve grafting is the gold standard for repairing a long-segment nerve defect [2]. Autologous nerve grafts are harvested typically from the sural nerve, the superficial branch of the radial nerve, the medial and lateral anterior cutaneous nerves of the arm, and the intercostal nerves [3]. This approach has some inherent disadvantages, such as limited autologous nerve supply, donor site morbidity (neuroma and pain), mismatch of donor and recipient nerve size or nerve fibre arrangement, and the risk of secondary surgery [4–6]. These issues have led to extensive investigation of alternative repair strategies, including nerve allografts [7], autologous venous catheter grafts [8], and tissue-engineering nerve conduits [9–11]. Of these, tissue-engineering nerve conduits are the most promising, offering several advantages, such as unlimited sources, customizable sizes and configurations, and personalized functionality by setting different loads [12]. 

The core elements of the tissue engineering nerve conduit are scaffold materials and seed cells. As a natural polymer, chitosan is widely used in gene delivery, cell culture, and tissue engineering due to its low toxicity, good histocompatibility, and biodegradability [13]. The United States Food and Drug Administration has approved nerve catheters made from chitosan materials for clinical use [14]. Seed cells and their derivatives are critical components of tissue-engineering, releasing various bioactive substances, providing a suitable microenvironment for nerve regeneration, guiding and promoting axon regeneration [15]. Schwann cells (SCs) and various types of stem cells have been shown to act as seed cells in tissue engineering nerve conduits, actively promoting nerve regeneration [16, 17]. However, SCs are end-stage cells that are difficult to expand in vitro and have limited clinical application. Among all types of stem cells, adipose-derived mesenchymal stem cells (ADSCs) are not only abundant in source, easy to obtain, and fast in proliferation rate, but also have a robust pro-angiogenesis function and few ethical restrictions, so they are widely used in clinical practice [18]. Studies have shown that ADSCs can promote nerve regeneration through the secretion of cytokines and growth factors or immunomodulatory effects [19, 20]. 

In the practical application of tissue engineering nerve conduits, the seed cells are primarily cultured in the traditional two-dimensional (2D) manner, that is, cells are injected into the nerve catheter and then cultured in a single layer. Although ADSCs can resist hypoxia and oxidative stress, the loss of cell suspension and the limited living space in the catheter may lead to an insufficient basic amount of ADSCs and poor transplantation efficacy [21]. Microcarriers are usually three-dimensional (3D) particles with a diameter of 50–400 μm, made of polysaccharide, protein, or polymer materials. They are widely used in the field of tissue engineering as carriers of drugs, cells, or growth factors [22]. The pores of microcarriers give them the characteristics of small volume and large relative surface area, which can provide good attachment points for cell growth, making them an ideal 3D cell culture system. In this way, cells and microcarriers can combine to form an excellent 3D microenvironment and improve the efficiency of cell expansion. Microcarrier-based cell transplantation strategies have been reported to play a beneficial role in bone and cartilage tissue defects [23], muscle repair [24], and skin wound healing [25]. However, there are few studies on using microcarrier complex cells in tissue engineering nerve conduits to treat peripheral nerve defects.

In this study, a novel tissue-engineered nerve graft was prepared by filling chitosan microcarriers loaded with ADSCs based on chitosan catheters. This study aimed to evaluate the role of this new tissue engineering nerve conduit in the reconstruction of sciatic nerve defects by systematically assessing its effect on nerve regeneration through gait analysis, electrophysiological testing, and histopathology of nerves and muscles.

## Methods

### In vitro study

#### Preparation and characterization of the hollow chitosan conduit

The preparation of the hollow chitosan conduit was performed as described previously [26]. Briefly, chitosan powder was dissolved in 2% (w/v) glacial acetic acid to form a 4% (w/v) chitosan solution. Air bubbles were removed to make the solution uniform. A specific mould was then dipped into the chitosan solution for a few seconds and slowly removed, the mould evenly coated with chitosan solution was frozen at -20 °C for 12 h and immersed in 2% (w/v) sodium hydroxide for 1 h to neutralise any remaining acetic acid and solidify the chitosan substrate. The chitosan tube was then carefully peeled from the mould, dried in a ventilated oven at 50 °C for 1 h and acetylated with acetic anhydride for 30 min. Finally, the hollow acetylated chitosan conduit was cut to a length of 14 mm and stored in 75% ethyl alcohol for further use.

The microstructures of the hollow chitosan conduit segments were observed with a scanning electron microscope (SEM, HITACHI-8010, Japan), and the samples were sputter-coated with a gold layer for 3 min under vacuum to increase conductivity.

### Preparation and evaluation of porous chitosan microspheres

As previously reported, porous chitosan microspheres were fabricated using a modified emulsion cross-linking method [27]. First, chitosan powder was dissolved in a 2% (v/v) aqueous acetic acid solution containing 0.9% (w/v) sodium chloride to obtain 2%(w/v) chitosan solution, which served as the aqueous phase (dispersed phase). Then, a surfactant span 80 was added dropwise to the paraffin liquid under continuous stirring to create a 2%(v/v) mixed solution, the oil phase (continuous phase). Subsequently, the aqueous phase was dropped slowly into the oil phase and stirred at 2000 rpm for 1 h at 4℃ to form a water-in-oil (w/o) emulsion, and the volume ratio of the aqueous phase to the oil phase was 1:10. The resulting emulsion was immediately poured into liquid nitrogen for quenching, and 25%(v/v) aqueous glutaraldehyde was slowly dropped into the w/o emulsion and stirred at a speed of 300 rpm for 1 h to crosslink chitosan droplets. It was then washed with − 20 ℃ petroleum ether to remove the paraffin liquid in the emulsion, filtered out with a filter sieve, and freeze-dried in a freeze-dryer for 24 h to get the porous chitosan microspheres. Finally, the microspheres were washed three times with ethyl alcohol to remove the remaining oil phase and another three times with deionized water to remove the adhering glutaraldehyde, and then lyophilized in a freeze dryer to obtain a free-flowing powder.

The morphological characteristics of the porous chitosan microspheres were determined using a scanning electron microscope (SEM, HITACHI-8010, Japan). The samples were coated with a layer of platinum to increase conductivity.

### Isolation and identification of cells

#### Isolation and identification of ADSCs

Primary ADSCs were isolated from the inguinal fat of 24 h old male SD rats [28]. Briefly, the rats were sacrificed, and the inguinal adipose tissue was isolated, minced into 1 mm^3^ pieces, and digested with collagenase II (1 mg/ml; Biosharp) dissolved in DMEM/F-12(Corning) for 30 min at 37 °C. Tissue suspension was centrifuged at 620×g for 10 min and filtered through a 70-µm sterile mesh to remove all tissue debris. Finally, the harvested cells were cultured in α-modified minimal essential medium (α-MEM; Hyclone) supplemented with 10% FBS and 1% penicillin/streptomycin solution. Cells at passages 2–4 were used for follow-up experiments.

Osteogenic, adipogenic, and chondrogenic stains were used to identify the characteristics of multidirectional differentiation capacity. For osteogenic and adipogenic differentiation, passage 2 ADSCs were seeded in six-well plates at a density of 2 × 10^5^ cells per well, and ADSCs were cultured for 21–28 days using osteogenic induction medium and adipogenic induction medium (Cyagen Biosciences). After induction, differentiated cells were washed with PBS, fixed with 4% paraformaldehyde, and stained with Alizarin Red S (an osteogenic marker) or Oil Red O working solution (an adipogenic marker). For chondrogenic differentiation, ADSCs at passage 2 were seeded at 5 × 10^5^ cells in a 15 ml sterile centrifuge tube and cultured with chondrogenic induction medium (Cyagen Biosciences) for 21 days. Chondrogenic pellets were fixed in 4% paraformaldehyde (Solarbio), cut into 5 μm thick paraffin sections, and the sections were stained with Toluidine Blue O working solution (Solarbio). Images were captured using an optical microscope (Leica, Wetzlar, Germany).

Flow cytometry was used to identify the surface markers of ADSCs. Briefly, ADSCs at passage 2 were labelled with anti-CD90 antibody (0.2 µg/10^6^ cells), anti-CD45 antibody (0.2 µg/10^6^ cells), anti-CD31 antibody (0.2 µg/10^6^ cells), and anti-CD29 antibody (0.2 µg/10^6^ cells) (BD Biosciences) in the dark at room temperature for 30 min. The characteristics of the ADSCs were then detected and analysed by flow cytometry (CytoFLEX; Beckman Colter Inc.).

### Isolation and identification of SCs

Primary SCs were extracted from the sciatic nerves of male SD rats at 24 h old. Briefly, the sciatic nerves, about 1 cm in length, were dissected from rats and removed the epineurium. The sciatic nerves were subsequently minced into small pieces measuring approximately 1 mm³. These pieces were then digested using 1 mg/ml collagenase NB4 (SERVA) and 0.025% trypsin for 15 min at 37 °C. After filtration, centrifugation, and resuspension, the SCs were seeded in DMEM/F12 medium supplemented with 2 mM forskolin (Sigma-Aldrich) and 2 ng/ml recombinant human NRG1-β1 (R&D system). The cells were incubated and when the SCs reached 80% confluence, they were purified from fibroblasts by digestion with collagenase NB4. Finally, purified SCs at passages 2–4 were utilized for subsequent experiments.

SCs were identified by staining with anti- S100 antibody (Sigma-Aldrich).

### Isolation and culture of rat dorsal root ganglions

Rat dorsal root ganglions (DRGs) were obtained from the intervertebral foramen of 12 h old male SD rats following the previous protocol [26]. The rats were sacrificed, then isolated their spines and cut into two halves along the central axis to expose DRGs fully. Removed the epineurium of DRGs, and cultured DRGs in DMEM/F-12 medium supplemented with 50 ng/ml of nerve growth factor (NGF), 10 µg/ml penicillin/streptomycin, 10% FBS, 2% B-27(Thermo Fisher Scientific), 2 mM glutamine (Thermo Fisher Scientific) in a humidified 5% CO_2_-containing atmosphere at 37 °C.

### Loading ADSCs on the porous chitosan microspheres

Before loading ADSCs onto the porous chitosan microspheres, the microspheres were sterilized by immersion in 70% ethanol for 6 h at 4 ℃, then the ethanol was replaced with deionized water and washed with PBS. For seeding, ADSCs were resuspended to a concentration of 1 × 10^5^ cells and added to culture media containing 20 mg of microspheres in a 12-well cell culture plate. The microspheres were evenly dispersed in the suspension, after which the culture plate was placed in the incubator for 6 h to ensure that the cells adhered to the microspheres. Then collected microspheres and rinsed with culture media to remove unattached cells, the cell-attached microspheres were resuspended with culture media and transferred to a new 12-well cell culture plate and cultured in the incubator.

### Live/dead staining

The biocompatibility of the microspheres was assessed by live/dead cell staining. Briefly, ADSCs-loaded microspheres were cultured for 1, 3, 5 and 7 days, then the cell medium was discarded and the ADSCs-loaded microspheres were washed with PBS. According to the specifications of the live/dead cell staining kit (Solarbio), cells were stained with calcein-AM (a green fluorescent dye for live cells) and propidium iodide (PI, a red fluorescent dye for dead cells). After staining, the ADSCs-attached microspheres were observed under a fluorescence microscope, and the number of the cells was determined semi-quantitatively according to the fluorescence intensity.

### Effects of 2D and 3D-cultured on ADSCs biological characteristics

#### Morphological observation

In this study, ADSCs were seeded on conventional cell culture plates or bottles, called 2D culture, and ADSCs loaded on porous chitosan microspheres, called 3D culture. ADSCs were cultured in both ways at 4 × 10^5^ cells per well for 5 and 7 days, after which the effect of the two types of culture on cell density was assessed using light microscopy. To further observe the cell morphology of 3D-cultured ADSCs, ADSCs were cultured on the porous chitosan microspheres for 3 days, stained with PKH26, and then examined with a confocal laser scanning microscope to examine the cells on the microspheres in a 3D view.

### Evaluation of cell secretion

To measure the concentration of vascular endothelial growth factor (VEGF) and transforming growth factor-β (TGF-β) of 3D-cultured ADSCs, the culture supernatants of the cells (4 × 10^5^) were collected after 1 day, 3 days, 5 days, 7 days and 9 days, and centrifuged at 1500 rpm at 4 °C for 10 min to remove the cell debris. The supernatants were then analysed by ELISA using Rat VEGF and TGF-β ELISA kits (Boster) according to the manufacturer’s instructions. The absorbance of each well was determined at 450 nm using a microplate reader (Labsystems Multiskan).

### Evaluation of cell proliferation

To evaluate the difference in proliferation between 2D and 3D cultured ADSCs. After 1 day, 3 days, 5 days and 7 days of 2D and 3D culture, the ADSCs were digested with 0.25% trypsin, centrifuged at 1000 × g for 5 min, and then resuspended in a single-cell suspension. The cell counter was used to count the number of ADSCs.

### 2D and 3D-cultured ADSCs were co-cultured with DRGs or SCs indirectly

#### DRG immunofluorescence

DRGs were indirectly co-cultured with 2D-cultured ADSCs or 3D-cultured ADSCs in a transwell system. Briefly, the lower chamber was seeded with DRGs, then 2D-cultured ADSCs and 3D-cultured ADSCs were seeded in the upper inserts, respectively. After co-culture for 5 days, immunofluorescence staining of S100 (primary antibody: Rabbit anti-S100 antibody, secondary antibody: Goat anti-rabbit IgG H&L (Alexa Fluor® 594)) and neurofilament protein 200 (NF200) (primary antibody: Mouse anti-Neurofilament 200 antibody; secondary antibody: Goat anti-mouse IgG H&L (Alexa Fluor® 488)) to stain the SCs and axon of the DRGs, and DAPI to stain the nuclei. Images were captured using a fluorescence microscope. Three DRGs from each group were randomly selected for statistical analysis. The two longest axons per quadrant were measured and used to calculate the mean axon length of each DRG using Image-Pro Plus software.

### SCs migration

The transwell assay was used to evaluate the effects of ADSCs cultured in two ways (2D culture and 3D culture) on the migration capacity of SCs. Briefly, 1 × 10^5^ SCs were seeded on the upper chamber with 8 μm pore filters, while 2D-cultured ADSCs or 3D-cultured ADSCs were added to the lower chamber at a density of 2 × 10^5^ cells per well. After incubation for 3 days, the SCs were fixed with 4% paraformaldehyde, and unmigrated cells on the upper chamber were gently wiped off with a cotton swab. The migrated cells were then stained with 1% crystal violet for 15 min. The relative number of migrated cells was counted and analysed in five randomly selected microscopic fields.

### In vivo study

This study has been reported in line with the ARRIVE guidelines 2.0.

### Animals

Male Sprague-Dawley (SD) rats (12 weeks old and weighing 250–300 g) were provided by Beijing Vital River Laboratory Animal Technology Co. Ltd, Beijing, China. Rats were housed in standard cages at room temperature (22 °C ± 2 °C) with a 12-hour light-dark cycle and free access to water and food.

### Surgical protocol

Forty-five SD rats were randomly divided into three groups (*n* = 15 per group) according to the different types of nerve grafts used to bridge sciatic nerve defects: (a) Hollow group, (b) Micro-S + ADSCs group, (c) Autograft group. All rats were anesthetized by intraperitoneal injection of 3% sodium pentobarbital solution (30 mg/kg). The posterolateral skin of the right thigh was sterilized with iodophor and the sciatic nerve was exposed. A 12 mm segment of the sciatic nerve was transected and removed with microsurgical scissors.

In the Micro-S + ADSCs group, the distal nerve stump was first sutured to the hollow chitosan conduit for 1 mm using 9 − 0 nylon monofilament suture, 50 µl of cell culture medium containing ADSCs-loaded porous chitosan microspheres (approximately 50 µg of porous chitosan microspheres by dry weight) was injected into the chitosan conduit, and the proximal stump was also sutured 1 mm into the hollow chitosan conduit, then the suture interfaces were sealed with fibrin gel. For the hollow group, 50 µl of cell culture medium without microspheres and ADSCs was injected into the chitosan conduit, while the other steps were the same as for the Micro-S + ADSCs group. For the autograft group, 12 mm of the sciatic nerve was cut, reversed, and reattached back to the nerve stumps. Finally, the muscles and skin were sutured after careful disinfection. All animals exhibited good postoperative recovery, with three animals experiencing transient abdominal distension for two days postoperatively, which resolved spontaneously.

### Gait analysis

Gait analysis was performed 12 weeks after transplantation to assess the motor recovery of all rats in each group. The CatWalk footprint analysis system (XT 10.6, Noldus) was used to record and analyse the rats’ footprints. Rats in each group walked on a glass-bottomed track, at least three approved runs were required for each rat in this experiment. A high-speed camera recorded the footprints, and the data were semi-automatically processed using the CatWalk XT 10.6 software. The software calculated the sciatic nerve function index (SFI) and the stand/swing time radio (SSR) for each rat.

#### Electrophysiological examination

An electrophysiological test was performed 12 weeks after transplantation to assess nerve conduction function. Rats were anesthetized, the previous surgical incision on the right lower limb was reopened, the sciatic nerve and gastrocnemius were exposed, and an electrophysiological instrument (Keypoint, Medtronic) was used to measure the compound muscle action potential (CMAP). Briefly, the stimulating electrode was placed 5 mm from the proximal end of the nerve conduits, and the recording electrodes were placed on the belly of the ipsilateral gastrocnemius muscle. CMAP amplitudes and latencies were elicited and recorded by electrical stimulation (3.0 mA, 1 Hz). CMAPs from the contralateral normal sciatic nerve were also recorded. The stimulus was repeated three times for each nerve in each rat.

### Histological evaluation of gastrocnemius

After electrophysiological evaluation, Masson’s trichrome staining was used to assess the histological recovery of the gastrocnemius muscle at 12 weeks after surgery. The gastrocnemius muscle of the operated hind limbs of rats in each group was harvested, and fixed in 4% paraformaldehyde for 2 h and embedded in paraffin. The gastrocnemius muscles were cut into 10 μm thick transverse sections and stained with a modified Masson’s trichrome staining kit (Solarbio). Images were captured under a microscope, and three visual fields were randomly selected from each sample and the percentage of collagen area and the mean cross-sectional area of gastrocnemius fibres were measured using Image-Pro Plus software.

#### Histological evaluation of nerve

At 3 weeks after transplantation, five rats in each group were sacrificed by overdose of sodium pentobarbital to observe the nerve growth at the early stage. Whole nerve grafts were harvested and fixed in 4% paraformaldehyde for 24 h, and 9 μm longitudinal frozen sections were obtained for immunofluorescence staining. The samples were washed in PBS, heterogeneous antigens were blocked with 10% goat serum for 30 min at room temperature and washed with PBS. The samples were then incubated overnight at 4 °C with primary antibodies against S100 (rabbit anti-S100 antibody; 1:200; Sigma-Aldrich) and NF200 (mouse anti-Neurofilament 200 antibody; 1:800; Sigma-Aldrich). The next morning, the samples were washed with PBS and incubated with secondary antibodies (goat anti-rabbit IgG H&L (Alexa Fluor® 594; 1:200; Abcam) and goat anti-mouse IgG H&L (Alexa Fluor® 488; 1:200; Abcam)) in the dark for 2 h at room temperature. After three washes with PBS, the nuclei were labelled with DAPI. Images were captured using a fluorescence microscope (Nikon Eclipse C1, Japan).

At 12 weeks post-transplantation, nerve grafts were carefully removed from all rats and the distal part of the nerve graft was carefully dissected to assess nerve regeneration. The nerve samples were cut transversely into 9 μm slices using a frozen slicer and immunofluorescence staining was performed as above. For toluidine blue (TB) staining, the samples were cut into transverse semithin sections of 1 μm thickness and stained with 1% toluidine blue solution (Solarbio); the stained sections were observed under a light microscope (EM UC7; Leica). For transmission electron microscopy (TEM) observation, the samples were cut into 70 nm transverse ultrathin sections, counterstained with 3% lead citrate and uranyl acetate, and observed by TEM (CM-120; Philips). For each sample in each group, three visual fields were selected to measure the mean diameter of the myelinated nerve fibres and the mean thickness of the myelin sheath using Image-Pro Plus software.

### Statistical analysis

Statistical analysis was performed using GraphPad Prism 9.5 and SPSS 22.0. The Kolmogorov-Smirnov test was used to examine the normal distribution of the data set. One-way ANOVA was used to compare differences in groups. Tukey’s multiple comparison *post hoc* test was applied when *p* > 0.05 in the test of homogeneity of variances, otherwise, Dunnett’s T3 post hoc test was applied. Statistical significance was defined as *p* < 0.05.

## Results

### Morphological characterization

#### Characteristics of hollow chitosan conduit

**Fig. 1:**
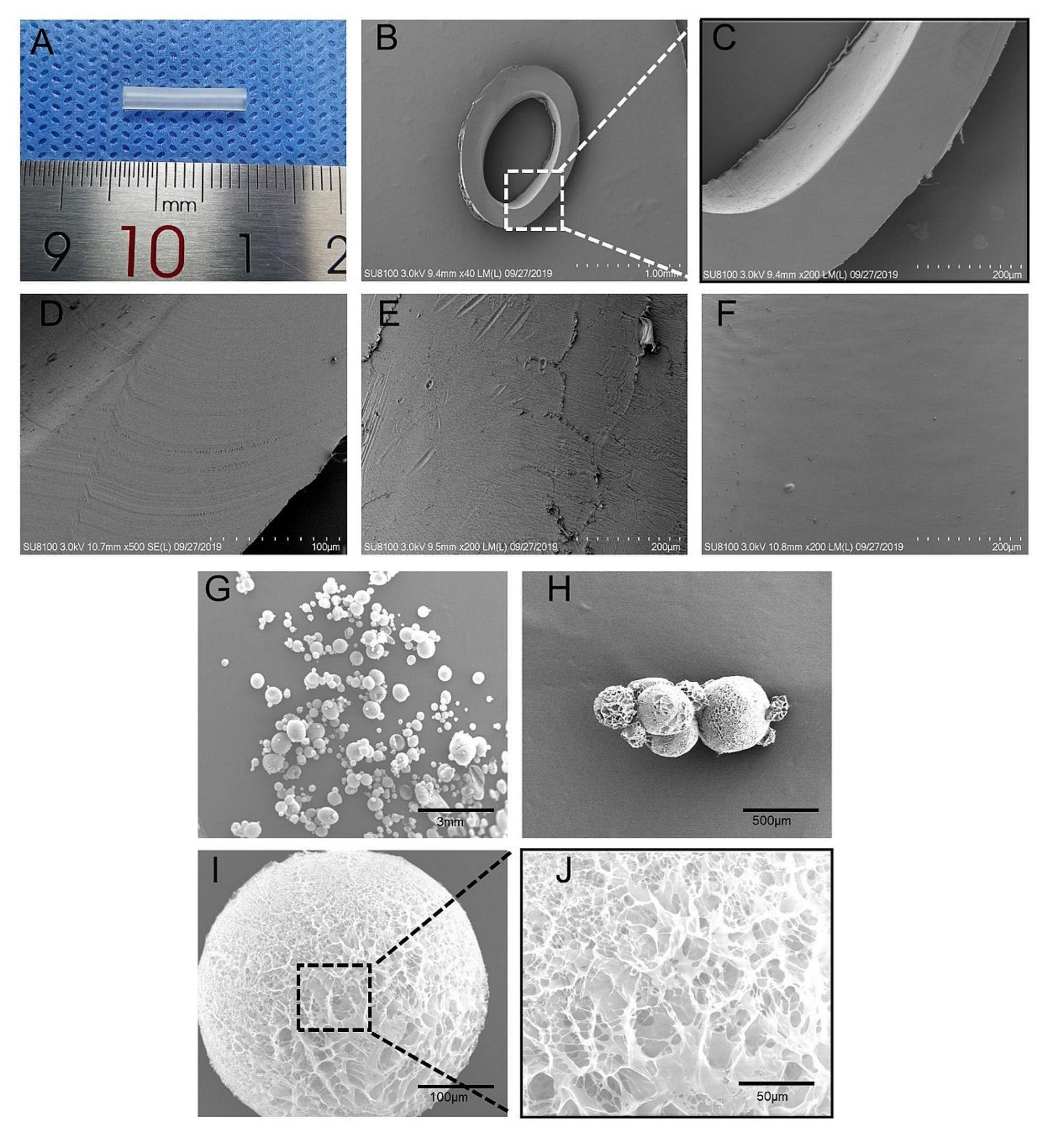
Characterization of hollow chitosan conduit and porous chitosan microspheres. **(A)** Gross image of hollow chitosan conduit. **(B)** SEM image of hollow chitosan conduit transverse section. Scale bar: 1 mm. **(C)** Magnified SEM image of hollow chitosan conduit transverse section. Scale bar: 200 μm. **(D)** SEM image of lateral surface morphology of hollow chitosan conduit. Scale bar: 100 μm. **(E)** SEM image of inner surface morphology of hollow chitosan conduit. Scale bar: 200 μm. **(F)** SEM image of outer surface morphology of hollow chitosan conduit. Scale bar: 200 μm. **(G-J)** SEM images of microspheres at different magnifications. Scale bar: 3 mm; 500 μm; 100 μm; 50 μm

Hollow chitosan conduits can be cut into different lengths as required. In this study, the length of hollow chitosan conduits used for nerve bridging was 14 mm (Fig. 1A). As shown in Fig. 1B-F, the SEM images of the hollow chitosan conduit showed that the lateral, inner and outer surfaces of the conduits were smooth.

#### Characteristics of porous chitosan microspheres

The resulting porous chitosan microspheres were observed by SEM. As shown in Fig. 1G-I, the microspheres displayed a spherical shape. Figure 1J revealed the presence of many pores evenly distributed on the surface of microspheres. Previous studies have shown that porous textured surfaces can promote cell adhesion and growth [29]. 

### Phenotype identification of ADSCs and SCs

**Fig. 2:**
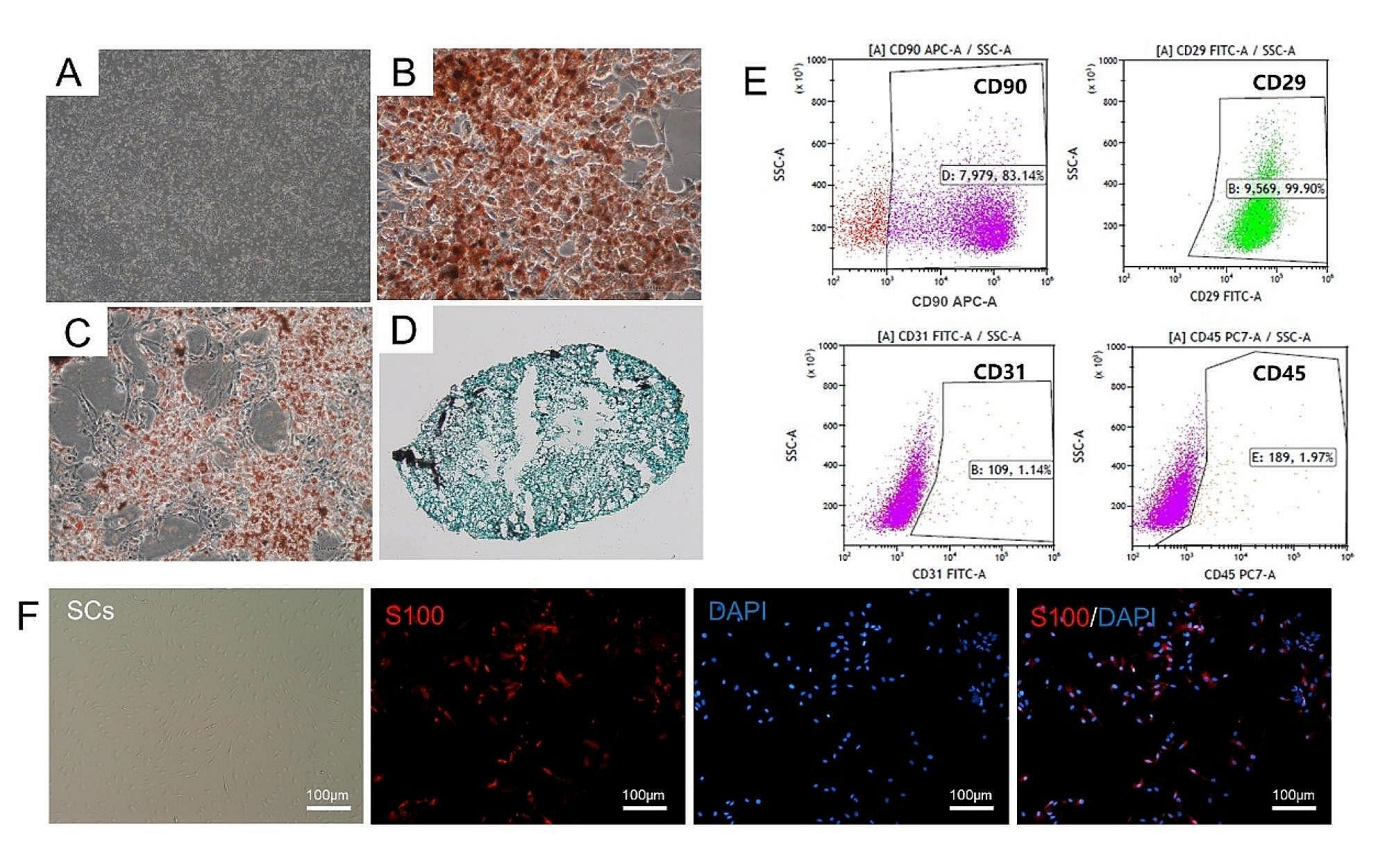
Identification of ADSCs and SCs. **(A)** The morphology of ADSCs under optical microscopy. Scale bar: 1 mm. **(B)** Alizarin Red S staining showed ADSCs differentiated into osteocytes formed red calcium nodules. Scale bar: 100 μm. **(C)** Oil Red O staining represented ADSCs differentiated into adipocytes filled with red lipid droplets. Scale bar: 200 μm. **(D)** Toluidine Blue O staining revealed that ADSCs differentiated into chondrocytes. **(E)** Flow cytometry analysis of surface maker ADSCs, the ADSCs were positive for CD90, CD29 but negative for CD31, CD45. **(F)** The morphology of SCs, SCs were immunolabeled by S100 (red), and nucleic acid was labelled by DAPI (blue). Scale bar: 100 μm

Optical microscopy images in Fig. 2A showed the ADSCs displayed a typical fibroblast-like morphology. After induction of differentiation, Alizarin Red S staining indicated that ADSCs could differentiate into osteoblasts with calcium deposits in the cells (Fig. 2B). Oil Red O staining evidenced that most cells could differentiate into adipocytes, forming red lipid droplets (Fig. 2C). Figure 2D demonstrated that ADSCs differentiated into chondrocytes with a typical rounded morphology. In Fig. 2E, flow cytometry showed that ADSCs were generally highly expressed in the stem cell markers CD90 (83.14%) and CD29 (99.90%), but were negative for the hematopoietic stem cell makers CD31 (1.14%) and CD45 (1.97%). These results indicated that the cells used in our experiments had the characteristics of ADSCs and a good capacity for differentiation. In Fig. 2F, the SCs exhibited a narrow fusiform shape with a bipolar or tripolar morphology, and the cells stained positive for S100, a well-established marker for SCs.

### Live/dead cell viability assay

**Fig. 3:**
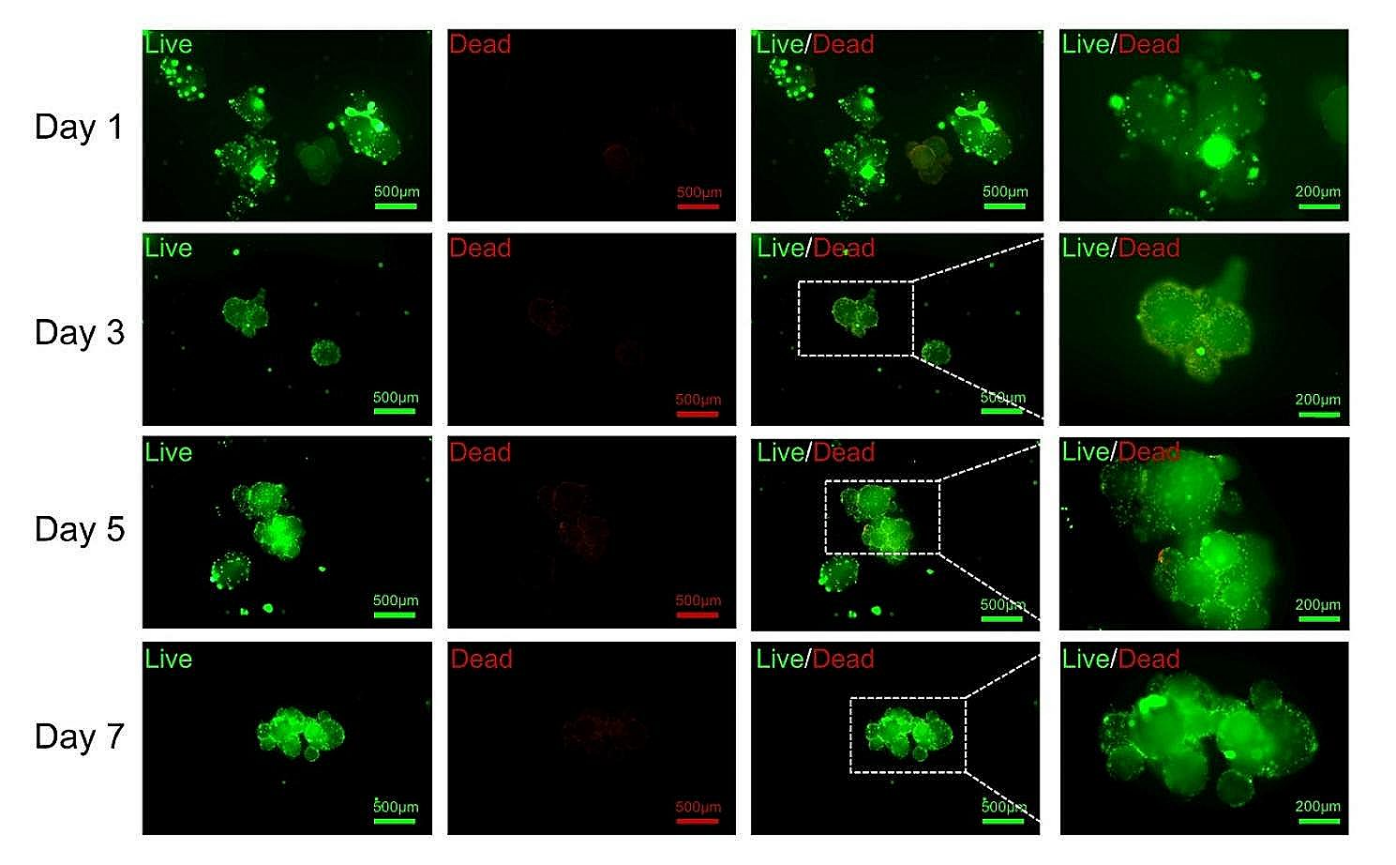
Live/dead staining images of ADSCs seeded on porous chitosan microspheres for 1, 3, 5, and 7 days. The live ADSCs were labelled with Calcein-AM (green). The dead ADSCs were labelled with PI (red). Scale bar: 500 μm and 200 μm

After routine culture of the ADSCs-loaded microspheres for 1, 3, 5, and 7 days, live/dead staining showed that cells were still alive over time, showing bright green fluorescence, while only a few dead cells showed dark red fluorescence (Fig. 3). The results confirmed the excellent cytocompatibility of porous chitosan microspheres.

#### Effects of 2D and 3D-cultured on ADSCs biological characteristics

**Fig. 4:**
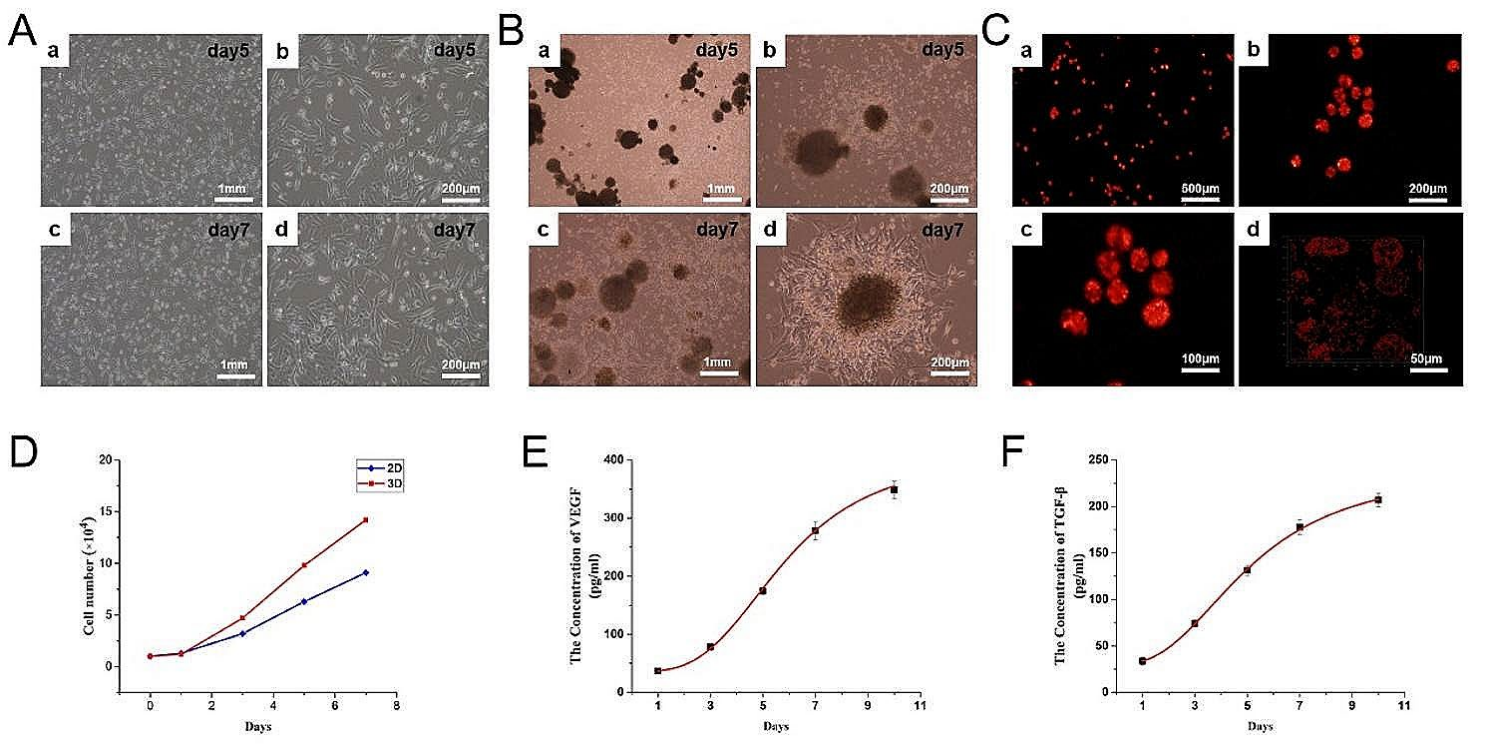
Effects of 2D and 3D-cultured on ADSCs biological characteristics. **(A)** Optical microscope photographs of ADSCs 2D cultured for 5 days and 7 days. Scale bar: 1 mm and 200 μm. **(B)** Optical microscope photographs of ADSCs 3D cultured for 5 days and 7 days. Scale bar: 1 mm and 200 μm. **(C) a-c** Confocal images of 3D-cultured ADSCs stained with PKH26. Scale bar: 500 μm, 200 μm and 100 μm; **d** 3D view of 3D-cultured ADSCs stained with PKH26. Scale bar: 50 μm. **(D)** Cell counting proved that the cell number of 3D-cultured ADSCs was more than 2D-cultured ADSCs. **(E-F)** VEGF and TGF-β secreted from 3D-cultured ADSCs were detected by Enzyme-linked immunosorbent assay. Data are expressed as mean ± SD

As shown in Fig. 4A and B, the cell densities on both ADSCs seeded on conventional culture plates (2D culture) and seeded on porous chitosan microspheres (3D culture) for 5 days were relatively low. After 7 days, more cells were observed on both ADSCs culture methods. Besides, Fig. 4D indicated that from day 3, the cell density of the 3D-cultured ADSCs was higher than that of the 2D-cultured ADSCs. In Figs. 4C, 3D-cultured ADSCs were labelled by PKH26 and observed with a confocal laser scanning microscope, and ADSCs were densely and uniformly packed on the porous chitosan microspheres. Figure 4E showed the levels of VEGF secreted by 3D-cultured ADSCs on day 1, day 3, day 5, day 7 and day 9, which showed a gradually increasing trend, and the levels of TGF-β secretion also showed a similar trend (Fig. 4F).

#### Effects of 2D and 3D-cultured ADSCs on DRGs and SCs

**Fig. 5:**
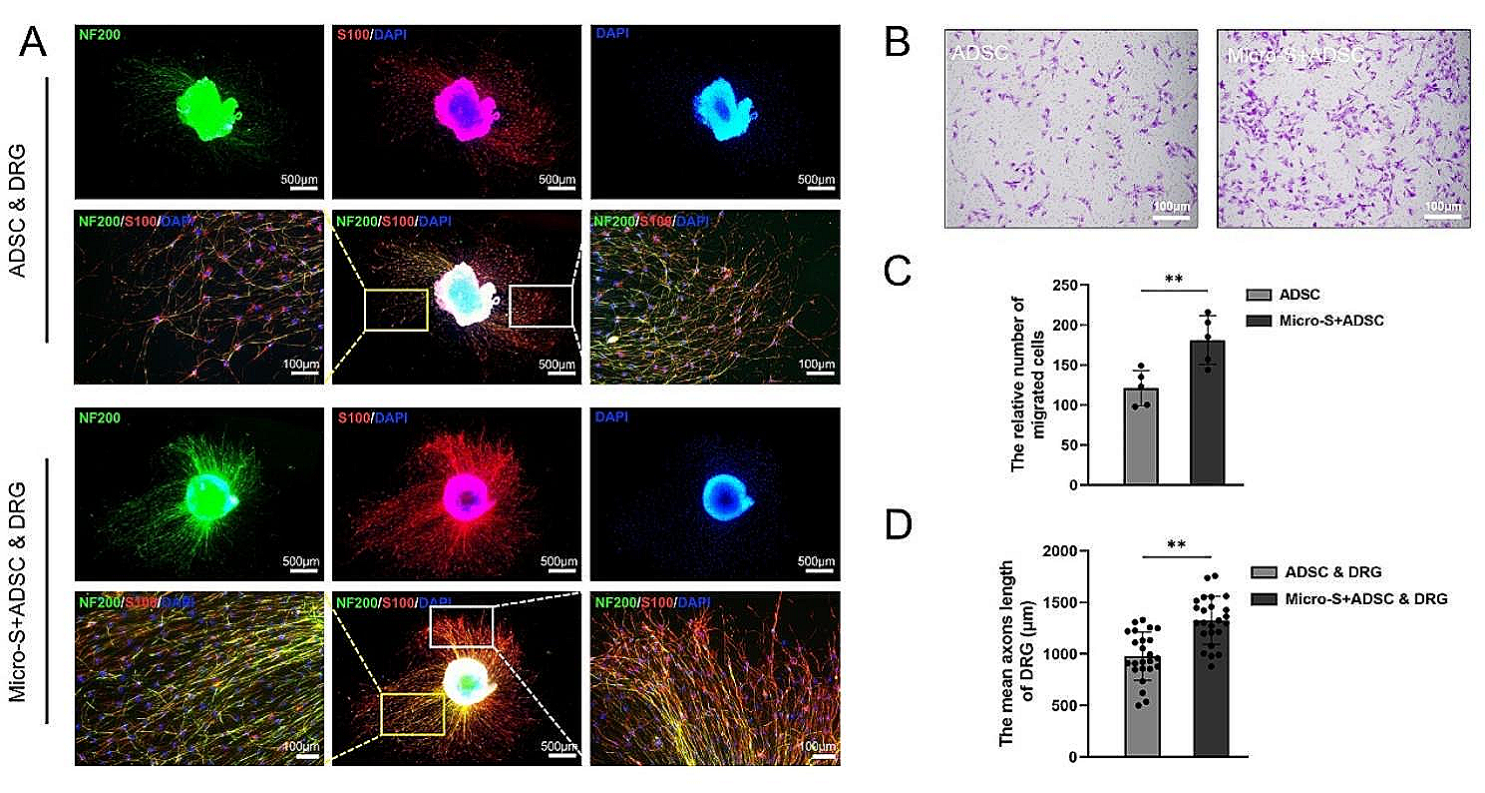
Effects of 2D and 3D-cultured ADSCs on DRGs and SCs. **(A)** Immunofluorescence staining images of DRGs co-culture with 2D or 3D-cultured ADSCs indirectly. The axons of DRGs exhibited green fluorescence, SCs that migrated from the DRGs exhibited red fluorescence, and all nuclei showed blue fluorescence. Scale bar:100 μm and 500 μm. **(B)** SCs migration was assessed using the Transwell assay. Scale bar:100 μm. **(C)** The relative number of migrated SCs in 2D and 3D-cultured groups. **(D)** The mean axon length of DRGs in 2D and 3D-cultured groups, Data are expressed as mean ± SD; ** *P*<0.01

To explore the difference in paracrine effects between 2D-cultured ADSCs and 3D-cultured ADSCs. DRGs and SCs were indirectly co-cultured with 2D or 3D-cultured ADSCs in transwell systems, respectively. Immunofluorescence staining showed that DRG axons in both groups stained green, SCs migrating from the DRG stained red and all nuclei showed blue fluorescence. The axon density of the DRG co-cultured with 3D-cultured ADSCs was higher (Fig. 5A). Further statistical analysis revealed that the mean axon length of DRG co-cultured with 3D-cultured ADSCs was also longer (Fig. 5D). Meanwhile, as shown in Fig. 5B and C, 3D-cultured ADSCs could also improve the migration ability of SCs.

### Recovery of motor function

**Fig. 6:**
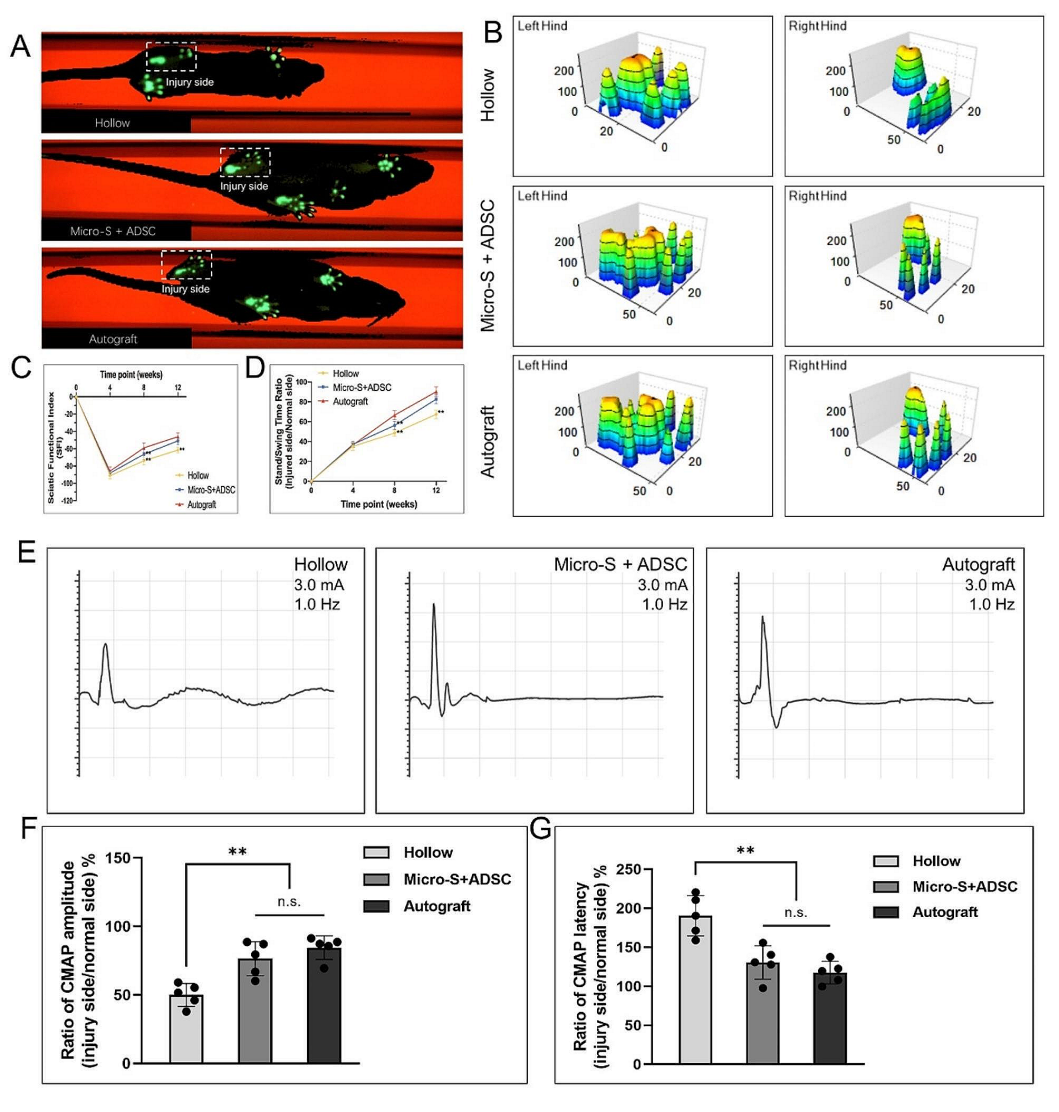
Gait analysis and electrophysiological examination at 12 weeks after transplantation. **(A)** Representative two-dimensional (2D) footprints images of the right (injured) hind and left (normal) hind paw of the hollow, Micro-S+ADSC and autograft groups **(B)** Typical three-dimensional (3D) stress diagrams of the left and right hands of each group. **(C)** SFI values from all of the groups. **(D)** Corresponding stand/swing time ratio of the three groups. *n* = 10 for each group. **(E)** Typical CMAP curves from the operated side of each group. **(F-G)** Quantification analysis of CMAP amplitude **(F)** and latency **(G)** (injury side/normal side). *n* = 5 for each group; Data are expressed as mean ± SD; **P < 0.01; n.s.: no significance

The motor functional recovery 12 weeks after implantation was examined by gait analysis. Representative footprint views of each group are shown in Fig. 6A, the intensity of pressure of the injury side leg contacted the floor in the Hollow group was markedly lower than that in the Micro-S + ADSC group, and the extension of the injury side toes in the Micro-S + ADSC group was improved than the Hollow group, and was closer to those in the Autograft group. According to the three-dimensional (3D) stress diagrams of each group shown in Fig. 6B, the stress area of the injury side of the Micro-S + ADSC group was larger than that of the Hollow group and similar to that of the Autograft group.

At the 4th week after transplantation, there was no statistical difference in SFI and Stand/Swing Time Ratio between the three groups. The SFI of the Autograft group was significantly higher than the Micro-S + ADSC group and Hollow group at the 8th week after transplantation (*P*<0.01, *P*<0.01). At the 12th week after transplantation, it is noteworthy that the SFI of the Micro-S + ADSC group was significantly higher than the Hollow group (*P*<0.01), and there was no statistical difference between the Micro-S + ADSC group and Autograft group (Fig. 6C). The trend of the Stand/Swing Time Ratio of the three groups at 4 weeks, 8 weeks, and 12 weeks after transplantation was the same as that of the SFI (Fig. 6D).

### Electrophysiological examination

At 12 weeks after the surgery, representative CMAP curves of four groups are shown in Fig. 6E. The ratio of CMAP amplitude and the ratio of CMAP latency were significantly improved in the Micro-S + ADSC and Autograft group compared with the Hollow group (*P*<0.01, *P*<0.01), and there was no statistical difference between Micro-S + ADSC and Autograft group (Fig. 6F-G).

### Histological evaluation of gastrocnemius

**Fig. 7:**
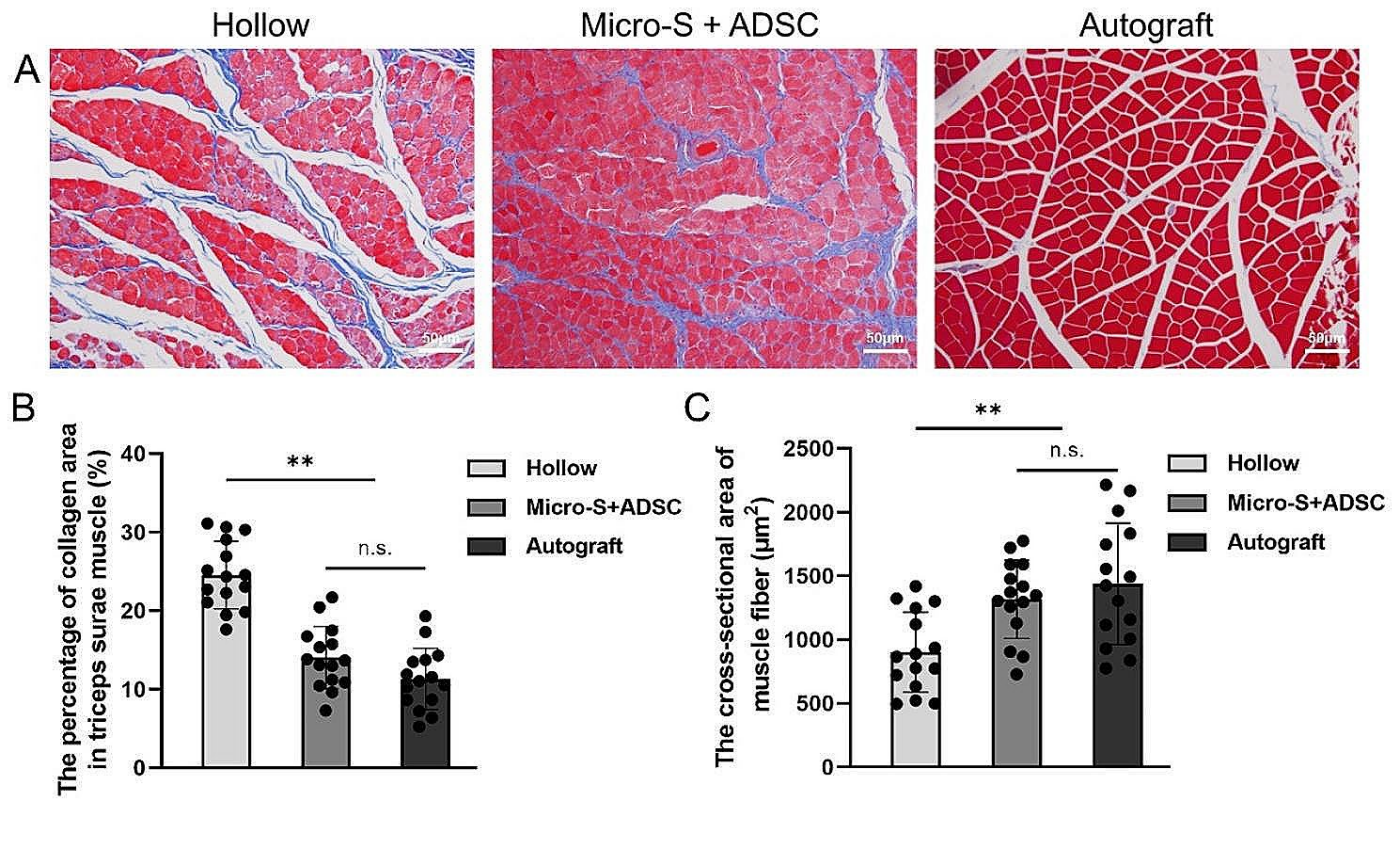
Histological evaluation of the gastrocnemius 12 weeks after surgery. **(A)** Masson’s trichrome staining images of the middle transverse sections of the gastrocnemius muscles in each group. Scale bar:50 μm. **(B-C)** Quantification analysis of the percentage of collagen area in the triceps surae muscle **(B)** and the cross-sectional areas of the muscle fibres **(C)**. *n* = 5 for each group; Data are expressed as mean ± SD; **P < 0.01; n.s.: no significance

At the 12th week after surgery, the target muscles of each group showed different degrees of atrophy, manifested as collagen fibre hyperplasia and muscle fibre area reduction. Masson staining of the gastrocnemius muscle in the three groups showed the hyperplasia of blue-stained collagenous between the red-stained muscle fibres (Fig. 7A). The percentage of collagen area in the triceps surae muscle in the Hollow group was significantly higher than the Micro-S + ADSC group and Autograft group (*P*<0.01, *P*<0.01), and there was no significant difference between the Micro-S + ADSC group and Autograft group (Fig. 7B). The cross-sectional areas of the muscle fibres in the Micro-S + ADSC group were significantly greater than the Hollow group (*P*<0.01), and there was no significant difference between the Micro-S + ADSC group and Autograft group (Fig. 7C).

#### Histological evaluation of regenerated nerves

**Fig. 8:**
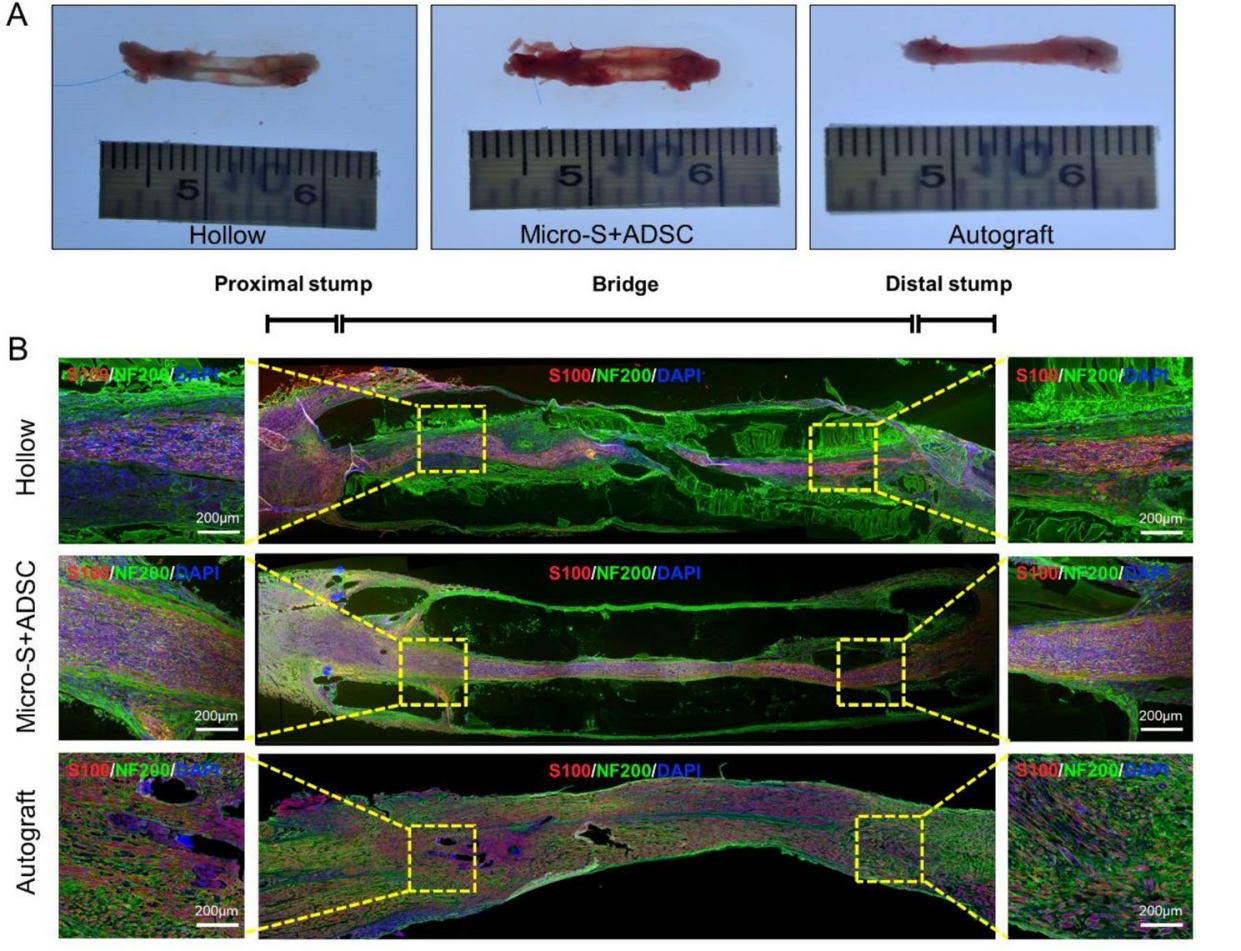
Histological evaluation of the nerve grafts at 3 weeks after transplantation. **(A)** Representative gross views of nerve grafts in the hollow, Micro-S+ADSC and autograft groups. **(B)** Immunofluorescence images of longitudinal sections of the regenerated nerves in three groups. The axons were stained with anti-Neurofilament 200 antibodies (green fluorescence), the myelin sheaths were stained with anti-S100 antibodies (red fluorescence), and all nuclei were stained by DAPI (blue fluorescence). Scale bar: 200 μm

To observe the growth of nerves at the early stage, nerve grafts from each group were obtained at the 3th week after transplantation and longitudinally cut into 9 μm slices for immunofluorescence staining. Gross images show that the nerve continuity of the Hollow group and Micro-S + ADSC group was restored, and the nerves of the Micro-S + ADSC group were coarser and had better regeneration (Fig. 8A). Immunofluorescence analysis showed that axons exhibited green fluorescence, the myelin sheath exhibited red fluorescence. Axonal growth was observed throughout the nerve graft in all three groups. However, the density of axons in nerve grafts of the Micro-S + ADSC group was higher than in the Hollow group and was closer to those in the Autograft group (Fig. 8B).

**Fig. 9:**
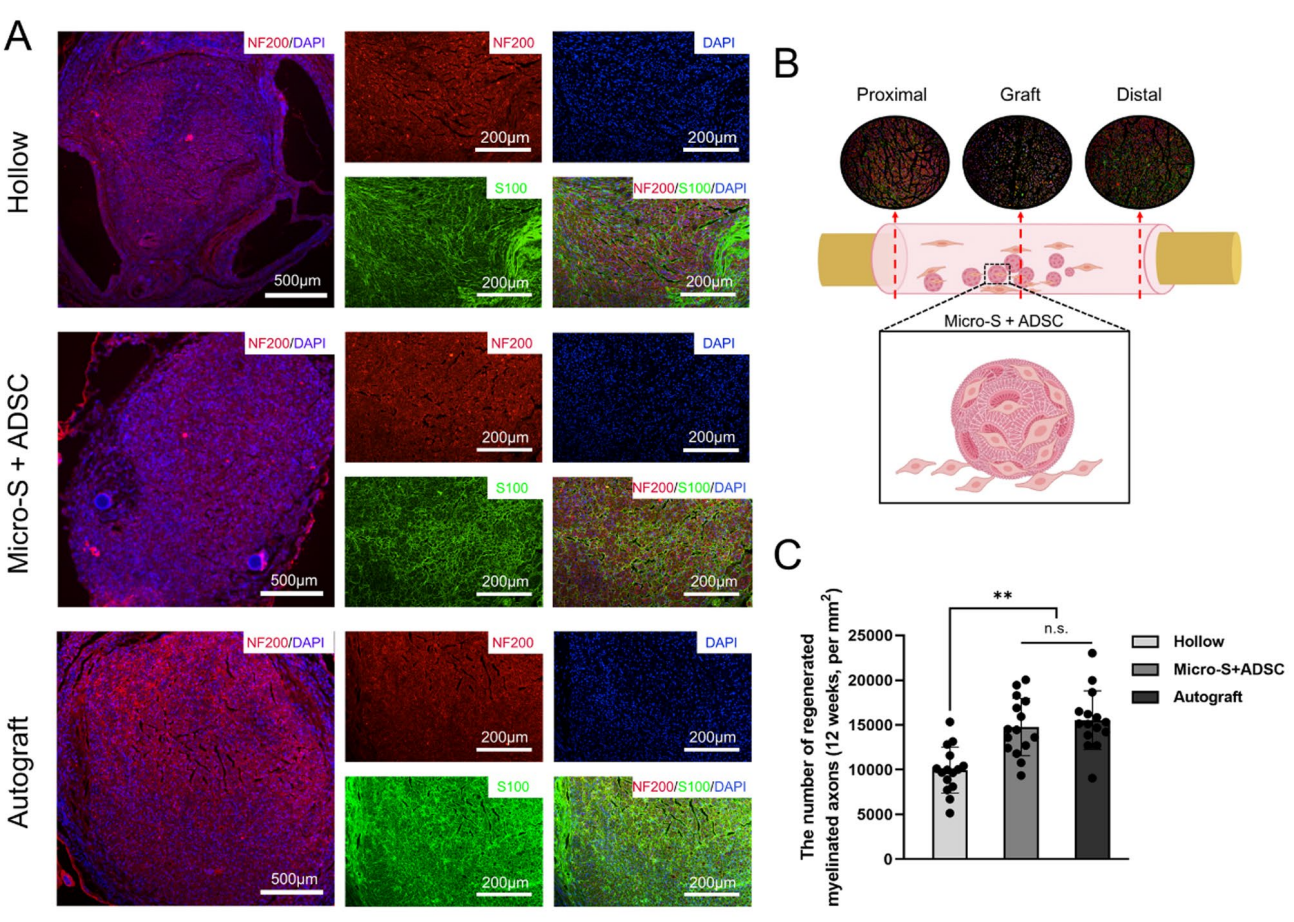
Histological evaluation of distal regenerated nerves at 12 weeks after transplantation. **(A)** Immunofluorescence photographs of transversal sections of the regenerated nerves in each group. The axons exhibited red fluorescence, the myelin sheaths showed green fluorescence, and all nuclei exhibited blue fluorescence. Scale bar: 500 μm and 200 μm. **(B)** Schematic diagram of proximal nerve, transplanted microspheres, and distal nerve at the site of nerve defect. **(C)** The number of regenerated myelinated axons (*n* = 5). Data are expressed as mean ± SD; **P < 0.01; n.s.: no significance

**Fig. 10:**
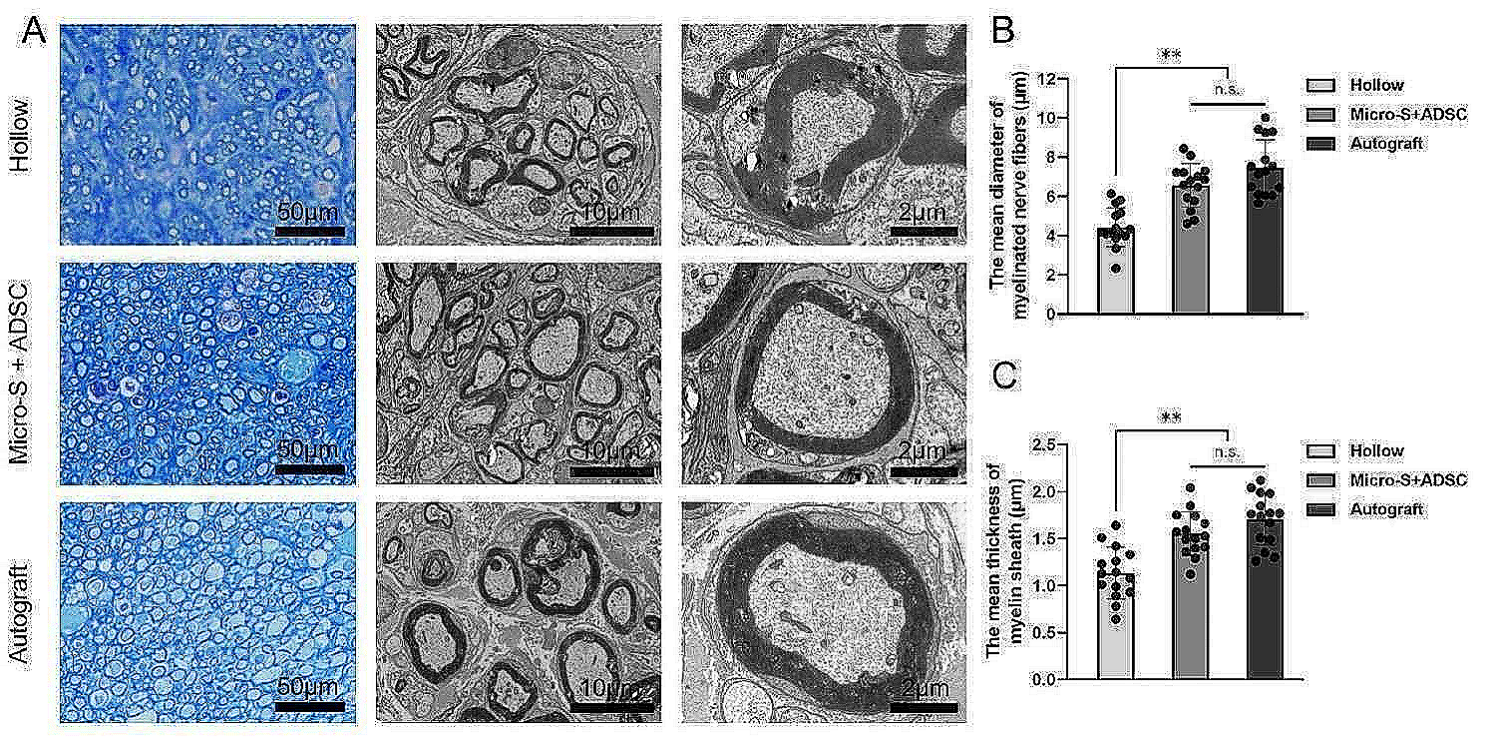
Evaluation of the regenerated myelinated nerve fibres in the distal portion at 12 weeks after surgery. **(A)** Toluidine blue staining and transmission electron microscopy (TEM) images of the regenerated sciatic nerve transverse sections. Scale bar: 50 μm; 10 μm; 2 μm. **(B)** The mean diameter of myelinated nerve fibres (*n* = 5 for each group). **(C)** Mean thickness of myelin sheath (*n* = 5 for each group). Data are expressed as mean ± SD; **P < 0.01; n.s.: no significance

At the 12th week after transplantation, immunofluorescence analysis of the distal segments of the grafts showed that axons exhibited red fluorescence, the myelin sheath exhibited green fluorescence (Fig. 9A). The number of regenerated myelinated axons in nerve grafts of the Micro-S + ADSC group was more than in the Hollow group and was close to those in the Autograft group (*P*<0.01) (Fig. 9C). Toluidine Blue staining and TEM results of regenerative nerves located at the distal section of the nerve graft showed that the mean diameter of myelinated nerve fibres and mean thickness of myelin sheaths in the Micro-S + ADSC group were significantly higher than those in the Hollow group (*P*<0.01, *P*<0.01), and there was no statistical difference between Micro-S + ADSC group and Autograft group (Fig. 10A-C).

## Discussion

The repair and functional reconstruction of defective peripheral nerve injury has always been a hotspot and difficulty in clinical medicine and basic research. Timely and effective treatment can improve the prognosis of patients and reduce the risk of disability. The results of this study indicate that porous chitosan microspheres can act as efficient 3D cell culture microcarriers for the transplantation and expansion of ADSCs. Adding chitosan microspheres loaded with ADSCs into synthetic chitosan catheters can effectively repair 12 mm sciatic nerve defect in rats and promote peripheral nerve regeneration.

Peripheral nerve injuries with no material loss or defects smaller than 3 mm can usually be repaired spontaneously or with a tension-free suture of the nerve stump [1]. However, when the continuity of the peripheral nerve is severely disrupted, and a large defect is present, the tension-free suture will not be possible, and appropriate grafts between the nerve stumps will be required to bridge the defect and support axonal regeneration [30]. With the advancement of regenerative medicine, especially tissue engineering, various types of tissue-engineered grafts show great potential as a repair method to supplement or replace autologous nerve transplantation. The use of nerve conduit combined with seed cells is an effective way to construct tissue engineering nerve grafts [15]. 

In the construction of tissue engineering nerve grafts, ensuring the efficiency and activity of seed cells is the key to playing a therapeutic role. ADSCs come from a wide range of sources and are easy to obtain and culture. With 1 g adipose tissue, 3.5 × 10^5^-1.0 × 10^6^ stem cells can be generated [31]. Moreover, ADSCs are excellent seed cells in tissue engineering due to their low senescence level and low immunogenicity. In the repair of peripheral nerve injury, ADSCs can secrete a variety of neurotrophic factors to promote axonal regeneration and sensory and motor function recovery, and can also secrete a large number of vascular endothelial growth factors to promote angiogenesis and angiogenesis in the injured area [32–34]. In addition, ADSCs also have neuroprotective, target organ protective, and immunomodulatory effects. Studies have shown that nerve conduits supplemented with ADSCs can repair nerve defects significantly better than alone [35]. 

Most ADSCs transplanted into nerve conduits are based on the traditional 2D-cultured method. However, studies have shown that 2D-cultured cells depend highly on the artificial growth environment and lack a self-secreted extracellular matrix, resulting in reduced cell viability [36, 37]. It has been reported that 3D-cultured conditions can aggregate extracellular matrix secreted by cells to form a suitable microenvironment conducive to cell proliferation and activity maintenance [38, 39]. The results of in vitro experiments in this study showed that the 3D porous chitosan microcarrier prepared could efficiently load ADSCs and maintain their activity. In addition, compared with 2D culture mode, ADSCs cultured in porous chitosan microcarriers had a significantly enhanced proliferation capacity and were also able to consistently secrete VEGF and TGF-β, which play an active role in the process of nerve regeneration and repair. SEM images showed that the prepared chitosan microcarriers appear as 3D spherical shapes with uniformly distributed micropores on the surface. This provides sufficient space for ADSCs to grow, which could effectively avoid the adhesion of cells during 2D culture, and make full use of the space of the nerve conduit, thereby improving the efficiency of cell transplantation.

SCs are essential structural and effector cells in the repair of peripheral nerve injury. After nerve injury, SCs migrate to form the Büngner belt, into which neurofilaments grow and guide the regeneration of axons, ultimately completing nerve repair, but their migration is slow after nerve injury [2, 40]. The results of this study demonstrated that microcarrier-based 3D-cultured ADSCs could significantly promote the migration of SCs and the elongation of DRG axons. In the in vivo experiments, we evaluated the effect of adding chitosan microcarriers loaded with ADSCs to nerve conduits on nerve regeneration in several ways.

The results showed that the regeneration effect of sciatic nerve myelin and axon in the Micro-S + ADSCs group was better than that in the Hollow group at 3 weeks after treatment. At 12 weeks after treatment, compared with the Hollow group, the regeneration of myelin and axon in the Micro-S + ADSCs group was more favourable, and the atrophy of the target muscle was less severe. The recovery of nerve conduction and motor functions was better, similar to that of the Autograft group. The results indicate that chitosan microcarriers can improve the transplantation efficiency of ADSCs to promote nerve regeneration better.

Since 1967, when the Dutch scholar Van Wezel developed the first microcarrier using DEAE-Sephadex A 50, the application of microcarriers began to develop rapidly at home and abroad, and microcarriers of various materials have been emerging [41, 42]. In the field of nerve repair, the materials used to prepare microcarriers include chitosan, collagen, fibrin, hyaluronic acid, alginate, and polyethylene glycol [43–46]. Among them, chitosan, widely found in nature and extracted from insects’ shells, is the product of chitin-N-deacetylation. Its good plasticity makes it commonly used to prepare 3D spherical microcarriers [26, 47]. The diameter, pore size, and porosity of the microcarriers can be adjusted according to demand by controlling the concentration of the liquid and oil phases, the volume ratio of the oil phase to the water phase, and the stirring rate [28, 48]. Chitosan microcarrier-loaded cells are effective for bone tissue engineering [49], skin tissue engineering [50], and tendon tissue repair [51]. In the repair of nerve injury, Li et al. [52] found that loading SCs with protein-modified chitosan microcarriers could effectively enhance peripheral nerve regeneration. Ao et al. [53] reported that loading SCs derived from bone marrow mesenchymal stem cells on chitosan microcarriers could effectively promote the recovery of sciatic nerve defects in rats. However, there are still few studies on chitosan microcarriers loaded ADSCs. In this study, ADSCs were loaded on the prepared chitosan porous microcarriers, and the results of live/dead staining showed that the ADSCs had good biocompatibility. Compared with conventional culture, ADSCs cultured on chitosan microcarriers have a faster expansion rate, and only new microcarriers need to be added for cell passage, which can avoid cell damage caused by trypsin digestion. Therefore, our study provides a novel and effective strategy for cell transplantation in tissue engineering nerve conduits.

This study has several limitations. Firstly, the effect of nerve repair was not compared with other forms of cell transplantation. Secondly, the ADSCs group and the Microspheres alone group were not included in the in vivo experiment. Lastly, the survival of ADSCs after transplantation and the effectiveness of different doses of ADSCs and microcarriers were not investigated. Further optimisation of the culture ratio of ADSCs to microcarriers based on the findings of this study could potentially improve the efficacy.

## Conclusion

In this study, we successfully fabricated porous chitosan microspheres for loading ADSCs and transplanted them into nerve conduits for repairing sciatic nerve defects in rats. The results showed that ADSCs could grow densely on the microcarriers and form cell spheres. ADSCs loaded on chitosan porous microcarriers had better therapeutic effects on axon regeneration, myelin regeneration, nerve regeneration, and motor function recovery and had good application potential in nerve repair.

## Data Availability

All data generated or analysed during this work are available from the corresponding author on reasonable request.
